# Baseline aortic pulse wave velocity is associated with central and peripheral pressor responses during the cold pressor test in healthy subjects

**DOI:** 10.14814/phy2.13357

**Published:** 2017-07-21

**Authors:** Anastasiya Borner, Kyle Murray, Claire Trotter, James Pearson

**Affiliations:** ^1^ Department of Biology University of Colorado Colorado Springs Colorado Springs Colorado

**Keywords:** Aortic pulse wave velocity, central pulse pressure, cold pressor test

## Abstract

Cold environmental temperatures increase sympathetic nerve activity and blood pressure, and increase the risk of acute cardiovascular events in aged individuals. The acute risk of cardiovascular events increases with aortic pulse wave velocity as well as elevated central and peripheral pulse pressures. The aim of this study was to examine the independent influence of aortic pulse wave velocity upon central and peripheral pressor responses to sympathetic activation via the cold pressor test (CPT). Twenty‐two healthy subjects (age: 18–73 years) completed a CPT with the left hand immersed in 2–4°C water for 3 min. During the CPT, central (from: 36 ± 7 to: 51 ± 12 mmHg) and peripheral pulse pressure increased (from: 54 ± 7 to: 66 ± 11; both *P *<* *0.05). In all subjects the increase in central pulse pressure during the CPT was independently associated with baseline aortic pulse wave velocity (*r* ^2^ = 0.221, *P *=* *0.027) but not age (*P *>* *0.05). In a subset of subjects with higher arterial stiffness, the increase in peripheral pulse pressure during the CPT was independently associated with baseline aortic pulse wave velocity (*r* ^2^ = 0.415, *P *=* *0.032) but not age (*P *>* *0.05). These data indicate that central and peripheral pulse pressure responses during sympathetic activation are positively and independently associated with aortic pulse wave velocity through a wide age range. Decreasing aortic pulse wave velocity in aged individuals with elevated arterial stiffness may help reduce the incidence of acute cardiovascular events upon exposure to cold environmental temperatures.

## Introduction

The risk of experiencing an acute cardiovascular event is elevated in aged individuals exposed to cold environmental temperatures (Danet et al. 1999; Abrignani et al. 2009; Wolf et al. 2009; Bhaskaran et al. 2010). Aged individuals experience greater increases in blood pressure, arterial stiffness (Hess et al. 2009; Wilson et al. 2010; Gao et al. 2012; Monahan et al. 2013) and sympathetic nerve activity (Greaney et al. 2014) relative to young individuals during whole body cold stress. These cold related pressor responses may further aggravate health risks in persons with cardiovascular diseases, such as hypertension (Keatinge et al. 1984; Mercer et al. 1999). Furthermore, the risk of incident cardiovascular events in aged individuals exposed to cold environments has been associated with increases in sympathetic nerve activity and consequent increases in blood pressure (Marchant et al. 1993; Culic 2007). Without simulating exposure to cold environmental temperatures, the cold pressor test increases sympathetic activity and blood pressure (Hines and Brown 1936; Victor et al. 1987; Seals 1990) thereby providing an opportunity to examine.

Aortic pulse wave velocity is a surrogate measure of arterial stiffness and is an independent predictor of acute cardiovascular event risk in a community population (Sutton‐Tyrrell et al. 2005; Mattace‐Raso et al. 2006; Willum‐Hansen et al. 2006; Mitchell et al. 2010). Increases in sympathetic nerve activity can acutely increase arterial stiffness (Boutouyrie et al. 1994; Swierblewska et al. 2010) and blood pressure during many different types of stressors (Lim et al. 2015; Maki‐Petaja et al. 2016). Baseline aortic pulse wave velocity increases through age (Mitchell et al. 2004; McEniery et al. 2005) and therefore, given its importance in predicting acute cardiovascular event risk, the consequence of an increased sympathetic nerve activation may be more significant for individuals with elevated arterial stiffness. Furthermore, central and peripheral pulse pressures are associated with incident cardiovascular events (Roman et al. 2007; Glasser et al. 2014). Central pulse pressure, for example, is a strong predictor of acute cardiovascular events including myocardial infarction over a 5 year period in individuals free of cardiovascular disease (Roman et al. 2007). Given this association with acute cardiovascular events, examining central and peripheral pressor responses to acute sympathetic stimulation via the cold pressor test throughout a wide range of age and aortic pulse wave velocities enables an insight into the influence of arterial stiffness upon the elevated cardiovascular event risk during cold exposure in aged individuals (Marchant et al. 1993; Culic 2007).

Arterial stiffness and central blood pressure increase in response to a cold induced stressor perturbation (Geleris et al. 2004; Edwards et al. 2006, 2008; Hess et al. 2009; Moriyama and Ifuku 2010; King et al. 2013; Hintsala et al. 2014; Lim et al. 2015) and this increase is related to baseline arterial stiffness in a young and an aged group (Hess et al. 2009). However, the association between baseline aortic pulse wave velocity and central blood pressure during a cold stressor in individuals representing a wide range of ages and arterial stiffness is unknown. Therefore, the aim of this study was to examine the influence of arterial stiffness, as indexed by aortic pulse wave velocity, upon the central and peripheral pressor responses to cold pressor test (Hines and Brown 1936) induced sympathetic activation (Victor et al. 1987; Seals 1990), in individuals spanning a wide range of chronological age (18–75 years) and arterial stiffness. We hypothesized that baseline pulse wave velocity would be an independent predictor of the pressor responses to the cold pressor test such that individuals with a higher pulse wave velocity would also demonstrate a higher central and peripheral pressor response.

## Materials and Methods

### Ethical approval

All subjects were informed of the purpose, procedures and risks of the study before providing their informed written consent. The Institutional Review Board at the University of Colorado Colorado Springs approved the protocol and consent. All procedures conformed to the standards set by the Declaration of Helsinki.

## Subjects

Twenty‐two subjects (eight females) participated in this study. Subject characteristics were; age 41 ± 19 years; height 175 ± 9 cm; weight 76.7 ± 18.1 kg (mean ± SD). Pre‐menopausal females were tested in the follicular phase of the menstrual cycle or the placebo phase, if they were taking birth control pills. Subjects were not taking cardiovascular acting medications (aside from birth control pills), were non‐smokers, were free of any known cardiovascular, metabolic, or neurological diseases and refrained from alcohol, caffeine, and exercise for 24 h before the study.

### Instrumentation and experimental protocol

Prior to instrumentation subject's height and weight were recorded. Arterial blood pressure was non‐invasively and continuously measured on a finger on the right hand using photoplethysmography (NexFin^®^, BMEYE, Amsterdam, Netherlands), corrected to the height of the heart and used to calculate mean arterial pressure (Bogert et al. 2010; Truijen et al. 2012; Ameloot et al. 2013). Heart rate was calculated from R‐R interval obtained from three lead electrocardiogram interfaced with a bioamp (ADInstruments, CO). Aortic pulse wave velocity (aPWV; m/sec) was estimated using an automated sphygmomanometer (Mobil‐O‐Graph^®^; I.E.M, Stolberg, Germany) (Weiss et al. 2012; Feistritzer et al. 2015) wrapped around the left arm. Applanation tonometry of the right radial artery was used to assess central pulse pressure and pulse wave reflection (SphygmoCor^®^, AtCor Medical, New South Wales, Australia). Central (ascending aortic) pressure waves were generated from the radial artery pressure waves using a validated and generalized transfer function (Pauca et al. 2001). Radial artery blood pressure waves were continuously stored in the data acquisition system and analyzed retrospectively via pulse wave analysis (SphygmoCor^®^) to estimate the central (aortic) blood pressure waveform. Using the integral software, central pulse pressure (CPP) was calculated as the difference between the systolic and diastolic aortic pressures, while central augmentation pressure (cAP) was calculated as the difference between the second and first aortic systolic pressure peaks. Augmentation index (Aix) was subsequently calculated as cAP expressed as a percentage of the CPP. Owing to alterations in heart rate throughout the cold pressor test (CPT), augmentation index was standardized to 75 bpm (AIx@75) (Wilkinson et al. 2000).

Following instrumentation, subjects rested in the supine position for 30 min to allow for the stabilization of fluid shifts. Baseline data was subsequently obtained. While remaining supine subjects completed the cold pressor test wherein the left hand was immersed up to the wrist in a bucket of water held between 0 and 4°C for 3 min resulting in robust increases in sympathetic nerve activity (Victor et al. 1987; Seals 1990). The water inside the bucket was carefully stirred throughout the cold pressor test.

### Data analysis

Hemodynamic data were collected continuously via a data‐acquisition system (Lab Chart, ADInstruments, CO). Baseline values of peripheral pressure and heart rate represent the average across a 60‐sec period. Baseline aPWV, AIx75 and central blood pressure variables represent the average of at least two successive measurements. Increases in all hemodynamic variables during the CPT are referenced as the value obtained at the point of peak change during the CPT. Data were continuously collected throughout the CPT and averaged at 12‐sec intervals to enable identification of the peak change in hemodynamic variables throughout the cold pressor test relative to baseline. To examine the influence of arterial stiffness upon the pressor responses to the cold pressor test we split subjects into either lower or higher arterial stiffness groups based upon the median aPWV (6.05 m/sec).

### Statistical analysis

Univariate regression was used to examine correlations between independent and dependent variables. Independent variables included age, height, weight, BMI and gender, as well as baseline hemodynamic variables (peripheral and central blood pressures, aPWV, AIx75 and heart rate). Dependent variables were the peak increases in peripheral and central blood pressures, AIx75 and heart rate during the CPT relative to baseline. Changes in hemodynamic variables during the CPT in all subjects and groups of lower and higher arterial stiffness were compared to baseline using a two‐way ANOVA (group × time). Non‐normally distributed variables were log transformed prior to analysis. A priori statistical significance set at *P *≤* *0.05. Data are reported as mean ± SD. Statistical analysis was performed using SPSS v24 (IBM, NY).

## Results

### All subjects

Descriptive data and baseline hemodynamic values are reported in Table 1. During the CPT, values for all measured hemodynamic variables, including central and peripheral pulse pressures, increased relative to baseline (all *P *<* *0.05; Table 1). In all subjects, univariate regression analysis indicated that neither height, weight, gender nor BMI were correlated with baseline aPWV, or any pressor variable either at baseline or at the point of greatest increase during the CPT (all *P *>* *0.05).

**Table 1:** Baseline and peak cardiovascular responses to the cold pressor test in all individuals and separated into low and high aPWV groups

	All (*n* = 22)		Lower Arterial Stiffness (*n* = 11)		Higher Arterial Stiffness (*n* = 11)	
	Baseline	CPT	Baseline	CPT	Baseline	CPT
Age (years)	41 ± 19 (18–73)	—	26 ± 7 (18–39)	—	56 ± 15^b^ (24–73)	—
Gender (Male/Female)	14/8	—	8/3	—	6/5	—
Height (cm)	175 ± 9	—	176 ± 10	—	173 ± 8	—
Weight (Kg)	76.7 ± 18.1	—	77.3 ± 14.4	—	76.2 ± 21.8	—
BMI (Kg/m^2^)	24.98 ± 4.47	—	24.69 ± 2.97	—	25.28 ± 5.74	—
aPWV (m/sec)	6.62 ± 1.74 (4.4–10.3)	—	5.25 ± 0.56 (4.4–6.0)	—	7.98 ± 1.40^b^ (6.1–10.3)	—
AIx75 (%)	6 ± 16	26 ± 10^a^	−2 ± 16	20 ± 12^a^	14 ± 13^b^	31 ± 5^a^^,^ c
Mean arterial pressure (mmHg)	90 ± 9	114 ± 9^a^	91 ± 10	114 ± 8^a^	90 ± 9	113 ± 11^a^
Peripheral systolic pressure (mmHg)	125 ± 10	152 ± 13^a^	127 ± 9	151 ± 13^a^	123 ± 11	153 ± 14^a^
Peripheral diastolic pressure (mmHg)	71 ± 9	89 ± 8^a^	72 ± 9	90 ± 7^a^	70 ± 9	88 ± 10^a^
Peripheral pulse pressure (mmHg)	54 ± 7	66 ± 11^a^	55 ± 6	64 ± 10^a^	53 ± 9	69 ± 12^a^
Central systolic pressure (mmHg)	109 ± 9	134 ± 13^a^	107 ± 10	135 ± 14^a^	110 ± 9	142 ± 11^a^
Central diastolic pressure (mmHg)	72 ± 9	91 ± 9^a^	73 ± 9	92 ± 8^a^	71 ± 9	90 ± 10^a^
Central pulse pressure (mmHg)	36 ± 7	51 ± 12^a^	34 ± 4	45 ± 9^a^	39 ± 8^b^	56 ± 12^a^^,^ c
Heart rate (bpm)	66 ± 9	77 ± 14^a^	69 ± 10	81 ± 17^a^	64 ± 7	77 ± 10^a^

AIx75, aortic augmentation index relative to 75 bpm. Values are means ± SD for 22 subjects. Range is included in parenthesis for age and aPWV. CPT, cold pressor test.

a Different from baseline within that respective group (*P *≤* *0.05).

b Different between lower and higher aPWV groups at baseline (*P *≤* *0.05).

c Different between lower and higher aPWV groups at peak increase during CPT (*P *≤* *0.05).

**Figure 1:**
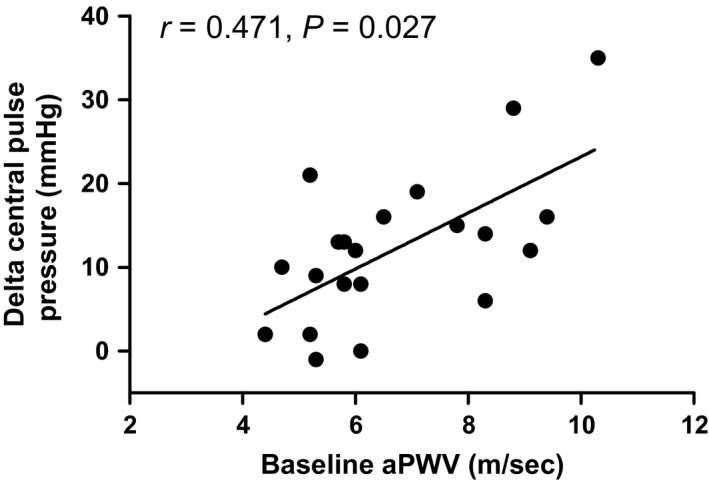
Correlation between baseline aortic pulse wave velocity and the increase in central pulse pressure during the cold pressor test in all subjects. The increase in central pulse pressure during the cold pressor test was independently predicted by aortic pulse wave velocity (*r* ^2^ = 0.221; *P *=* *0.027) but not age (*P *>* *0.05).

Relative to baseline central pulse pressure increased during the CPT (36 ± 7 vs. 51 ± 12 mmHg; *P *<* *0.05). Univariate regression analysis indicated that aPWV was the only independent variable correlated with the increase in central pulse pressure during the CPT (*r* ^2^ = 0.221, *P *=* *0.027; Fig. 1, Table 2). The increase in central pulse pressure during the CPT was not correlated with baseline central pulse pressure (*r* ^2 ^= 0.096, *P *=* *0.160). The increase in peripheral pulse pressure during CPT from baseline (from: 54 ± 7 to: 66 ± 11; *P *<* *0.05) was correlated with aPWV (*r* ^2^ = 0.423, *P *=* *0.001) and age (*r* ^2^ = 0.274, *P *=* *0.012).

**Table 2:** Univariate regression analysis results for the correlation between age, baseline aPWV and baseline AIx75 with the change in central and peripheral pressures during the CPT

	Age (years)	Baseline aPWV (m/sec)	Baseline AIx75 (%)
All subjects			
Peripheral pulse pressure (mmHg)	*r* = 0.523	*r* = 0.650	*r* = 0.317
	*r* ^2^ = 0.274	*r* ^2^ = 0.423	*r* ^2^ = 0.101
	*P* = 0.012^a^	*P* = 0.001^a^	*P *=* *0.150
Central pulse pressure (mmHg)	*r* = 0.369	*r* = 0.470	*r* = 0.125
	*r* ^2^ = 0.093	*r* ^2^ = 0.221	*r* ^2^ = 0.016
	*P* = 0.091	*P* = 0.027^a^	*P *=* *0.581
Lower arterial stiffness			
Peripheral pulse pressure (mmHg)	*r* = 0.184	*r* = 0.446	*r* = −0.148
	*r* ^2^ = 0.034	*r* ^2^ = 0.199	*r* ^2^ = 0.022
	*P* = 0.588	*P *=* *0.169	*P *=* *0.663
Central pulse pressure (mmHg)	*r* = 0.243	*r* = 0.350	*r* = −0.225
	*r* ^2^ = 0.059	*r* ^2^ = 0.122	*r* ^2^ = 0.050
	*P* = 0.472	*P *=* *0.292	*P *=* *0.507
Higher arterial stiffness			
Peripheral pulse pressure (mmHg)	*r* = 0.514	*r* = 0.644	*r* = 0.385
	*r* ^2^ = 0.264	*r* ^2^ = 0.415	*r* ^2^ = 0.148
	*P* = 0.106	*P *=* *0.032^a^	*P *=* *0.243
Central pulse pressure (mmHg)	*r* = 0.180	*r* = 0.385	*r* = 0.135
	*r* ^2^ = 0.032	*r* ^2^ = 0.149	*r* ^2^ = 0.018
	*P* = 0.597	*P* = 0.242	*P *=* *0.691

AIx75, aortic augmentation index relative to 75 bpm. Results are representative of data from 22 subjects.

a Relationship statistically significant (*P *≤* *0.05).

### Increased arterial stiffness

Descriptive subject data, baseline and peak increases in hemodynamic as well as central and peripheral pressures during the CPT are reported in Table 1 for higher and lower arterial stiffness groups. Central systolic and diastolic pressures, peripheral pressure variables, heart rate, height, weight and BMI were not different at baseline between groups (all *P *>* *0.05). However, in the higher arterial stiffness group, age (56 ± 15 years), aPWV (7.98 ± 1.40 m/sec), baseline AIx75 (14 ± 13%) and baseline central pulse pressure (39 ± 8 mmHg) were greater relative to the lower arterial stiffness group (26 ± 7 years, 5.25 ± 0.56 m/sec, −2 ± 16% and 34 ± 4 mmHg, respectively; all *P *<* *0.05).

**Figure 2:**
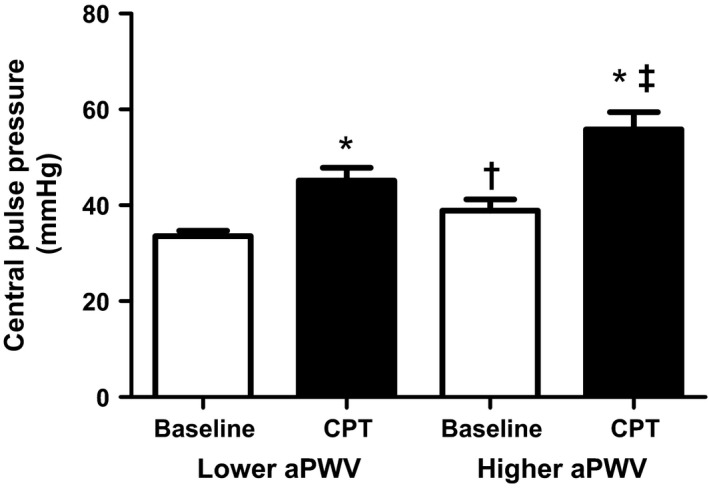
Peripheral and central pulse pressures at baseline and during the CPT in lower and higher arterial stiffness groups. Peripheral and central pulse pressures increased during the CPT in both groups. The central pulse pressure was greater during the CPT in the group with higher arterial stiffness. *Different from baseline within that respective group. †Different between lower and higher aPWV groups at baseline. ‡Different between lower and higher aPWV groups at peak increase during CPT. CPT, cold pressor test

**Figure 3:**
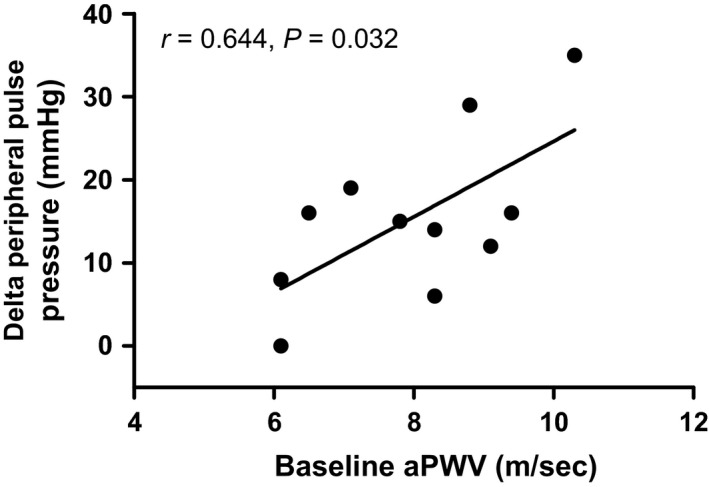
Correlation between baseline aortic pulse wave velocity and the increase in peripheral pulse pressure during the cold pressor test in subjects with higher arterial stiffness. In individuals with higher arterial stiffness the increase in peripheral pulse pressure during the cold pressor test was independently predicted by aortic pulse wave velocity (*r* ^2^ = 0.415; *P *=* *0.032) but not age (*P *>* *0.05).

Peripheral and central pressures, heart rate and AIx75 increased during the CPT relative to baseline in both groups (all *P *<* *0.05; Table 1; Fig. 2). In the higher arterial stiffness group the increase in AIx75 (to 31 ± 5%) and central pulse pressure (to 56 ± 12 mmHg) during the CPT was greater relative to the lower arterial stiffness group (to 20 ± 12% and to 45 ± 9 mmHg, respectively; both *P *<* *0.05). In both groups, the increase in central and peripheral pressures during the CPT did not correlate with height, weight, BMI, gender or age (all *P *>* *0.05). Of all measured independent variables, the increase in peripheral pulse pressure in the higher arterial stiffness group was only correlated with baseline aPWV (*r* ^2^ = 0.415, *P *=* *0.032; Fig. 3; Table 2). In the higher arterial stiffness group, the increase in central pulse pressure during the CPT did not correlate with any of the independent variables measured.

## Discussion

The aim of this study was to examine the influence of arterial stiffness upon the central and peripheral pressor responses to acute sympathetic activation via cold pressor test, in individuals spanning a wide range of age and arterial stiffness. Across this range, aortic pulse wave velocity correlated with the increase in central pulse pressure during the CPT while age did not. In individuals with an elevated arterial stiffness, aortic pulse wave velocity was correlated with the increase in peripheral pulse pressure during the CPT while age did not share a correlation with any pressor response. These data indicate that baseline arterial stiffness, as indexed here by aortic pulse wave velocity, is an independent predictor of the central and peripheral pressor responses to acute sympathetic activation via the cold pressor test.

We chose to examine the association between the acute central and peripheral pressor responses with baseline arterial stiffness during a cold pressor test as this perturbation increases sympathetic nerve activity and blood pressure (Greene et al. 1965; Victor et al. 1987). Increased sympathetic activity and blood pressure have been associated with the increased incidence of acute cardiovascular events occurring in aged individuals exposed to cold environmental temperatures (Marchant et al. 1993; Culic 2007). Moreover, central and peripheral pulse pressures are independent risk factors for incident cardiovascular events (Roman et al. 2007; Glasser et al. 2014). Previous studies have reported similar increases in central and peripheral pressor responses to cold induced stressors to those reported in this study (Geleris et al. 2004; Edwards et al. 2006, 2008; Hess et al. 2009; King et al. 2013; Hintsala et al. 2014; Lim et al. 2015). A relationship between resting arterial stiffness and the pressor response to whole body cold stress has been reported in a subject population comprised of young and aged individuals (Hess et al. 2009), which also displayed distinct differences in baseline arterial stiffness between these two groups. Here, we examined the association between baseline arterial stiffness and the acute central and peripheral pulse pressure responses to the cold pressor test across a wide range of baseline arterial stiffness and age. Our aim was to gain an insight into the influence of increased arterial stiffness through aging upon these cardiovascular risk factors during acute increases in sympathetic activation via the cold pressor test. These data indicate that increases in central pulse pressure during the cold pressor test were correlated with baseline aortic pulse wave velocity but not age in a group of individuals spanning a wide range of age and baseline arterial stiffness (Fig. 1). When individuals were split into either lower or higher arterial stiffness groups based upon aortic pulse wave velocity, the rise in central pulse pressure during the CPT was greater in the higher arterial stiffness group (Table 1), although it did not correlate with either age or arterial stiffness (Table 2). However, in the higher arterial stiffness group, aortic pulse wave velocity was correlated with the increase in peripheral pulse pressure during the CPT (Fig. 3; Table 2) while age was not correlated with any pressor response. Taken together, the acute central and peripheral pulse pressure responses to acute sympathetic activation are greater in individuals with a higher aortic pulse wave velocity and are independent of age.

It is important to note that the positive correlations between arterial stiffness and increased central and peripheral pulse pressures during the CPT were independent of chronological age. Arterial stiffness was indexed by aortic pulse wave velocity, a measure of central vascular aging. Acute increases in arterial stiffness during sympathetic activation are likely of particular importance to individuals with an already elevated central and peripheral pulse pressure given the association with incident cardiovascular events (Roman et al. 2007; Glasser et al. 2014). We chose to examine the relationship between baseline aortic pulse wave velocity and the pressor responses during CPT, rather than its increase during the CPT, because baseline aortic pulse wave velocity is an independent predictor of cardiovascular events (Sutton‐Tyrrell et al. 2005; Mattace‐Raso et al. 2006; Willum‐Hansen et al. 2006; Mitchell et al. 2010). Furthermore, while aortic pulse wave velocity increases through age (Mitchell et al. 2004; McEniery et al. 2005) it is lower in individuals with a history of lifelong habitual exercise training relative to sedentary counterparts (Vaitkevicius et al. 1993; Tanaka et al. 1998; Gates et al. 2003; Pierce et al. 2013, 2016) and can be reduced with acute exercise‐training even in aged, sedentary and diseased individuals (Tabara et al. 2007; Madden et al. 2009; Corrick et al. 2013; Tanaka et al. 2013; Vogel et al. 2013; Donley et al. 2014). Therefore, while chronological aging and increasing arterial stiffness generally share a close relationship there is likely between subject variations in this relationship. Within the context of the results of this study, a reduction in aortic pulse wave velocity would attenuate the rise in central and peripheral pulse pressures during acute sympathetic activation. Central aortic pressure is indicative of the pressure exerted upon central organs such as the heart (McEniery et al. 2014), and a greater central pulse pressure, for example, is likely reflective of an increased left ventricular afterload and coronary artery pressure (Roman et al. 2007), which may lead to impaired left ventricular diastolic function (Subherwal et al. 2010). In line with this, elevated central and peripheral pulse pressures are a risk factor for incident cardiovascular events, including myocardial infarction (Roman et al. 2007; Glasser et al. 2014). Therefore, a greater increase in pulse pressure upon acute sympathetic activation will likely exacerbate this incident cardiovascular risk. The influence of aortic pulse wave velocity upon the augmented central and peripheral pulse pressure responses to sympathetic activation found here, which was independent of chronological age, may help us better understand the increased incidence of acute cardiovascular events during cold temperature exposure in aged individuals (Danet et al. 1999; Abrignani et al. 2009; Wolf et al. 2009; Bhaskaran et al. 2010).

Aortic pulse wave velocity independently explained 22% of the peak increase in central pulse pressure during the CPT in all individuals, and 42% of the increase in peripheral pulse pressure during the CPT in individuals with a higher arterial stiffness. Therefore, aortic pulse wave velocity did not explain all the variance in the central and peripheral pressor responses to acute sympathetic activation via the CPT. Some of this additional variance may owe to sympathetic nerve activity, which is elevated in individuals with increased arterial stiffness (Boutouyrie et al. 1994; Swierblewska et al. 2010). If the increase in sympathetic activation was the same in all individuals during the CPT (Ng et al. 1994), sympathetic nerve activity may have been greater in individuals with higher versus lower aortic pulse wave velocity, owing to a higher baseline value. Additionally, aortic and carotid stiffening impair baroreceptor sensitivity (Monahan et al. 2001a,b; Mattace‐Raso et al. 2007). As central and peripheral pressures increased in all subjects during the CPT, it is possible that reduced baroreflex sensitivity impaired compensatory parasympathetic control in individuals with elevated arterial stiffness. Taken together, an augmented cardio acceleratory and/or vasoconstrictor effect during the CPT may have contributed to heightened central and peripheral pulse pressures during the CPT and blood pressure‐dependent rise in aortic pulse wave velocity (Maki‐Petaja et al. 2016; Harvey et al. 2017) in individuals with arterial stiffening. Nevertheless, while other factors undoubtedly contribute to the increased central and peripheral pulse pressures during acute sympathetic activation via the CPT, a significant proportion of this variability is associated with aortic pulse wave velocity and is independent of chronological age.

### Limitations

We utilized a cold pressor test to cause acute increases in sympathetic nerve activity allowing examination of the consequent central and peripheral pressor responses and their relationship with baseline aortic pulse wave velocity. While the cold pressor test is a different perturbation to cold stress and not representative of natural seasonal cold exposure, we employed it in this study to create a profound and robust increase in sympathetic nerve activity (Victor et al. 1987; Seals 1990) which has been hypothesized to accompany increased cardiovascular events upon exposure to cold temperatures (Marchant et al. 1993; Culic 2007). However, we did not measure sympathetic nerve activity in this study and therefore, we were unable to quantify the influence of sympathetic nerve activity upon the central and peripheral pressor responses. However, central and peripheral pressures increased in all subjects during the CPT (Table 1 and Fig. 2) and therefore it is reasonable to possible that the CPT resulted in physiologically significant increases in sympathetic nerve activity in all individuals. Second, increases in sympathetic nerve activity may have increased more in aged relative to young individuals, as shown during whole body cold stress (Greaney et al. 2014), but not a cold pressor test (Ng et al. 1994). Although sympathetic nerve activity was not measured here, given that this study employed the cold pressor test it is reasonable to hypothesize that the increase was similar across all individuals. Interestingly, the increases in central and peripheral pulse pressures observed here during the CPT (Table 1) were similar to those observed during whole body skin surface cooling in older individuals (Hess et al. 2009). These similarities suggest that alterations in central and peripheral pulse pressures during increased sympathetic activity may be comparable between a cold pressor test and skin surface cooling although further studies are required to clarify this. Furthermore, physical fitness and habitual activity influence vascular health (Vaitkevicius et al. 1993; Tanaka et al. 1998, 2013; Gates et al. 2003; Tabara et al. 2007; Madden et al. 2009; Corrick et al. 2013; Pierce et al. 2013, 2016; Vogel et al. 2013; Donley et al. 2014) while baroreflex sensitivity also changes with age and arterial stiffening (Monahan et al. 2001a,b; Pierce et al. 2016). We did not measure these and differences in these variables within our subject pool may have influenced the pressor responses to the cold pressor test. Finally, after splitting the subject population into lower and higher arterial stiffness categories, the sample size was lower relative to other studies examining the association of arterial stiffness with cardiovascular disease events.

### Perspectives and significance

These data indicate that aortic pulse wave velocity is a modest, but independent predictor of the central and peripheral responses during a cold pressor test and, presumably, activation of the sympathetic nervous system. Importantly the associations between aortic pulse wave velocity and pulse pressures were independent of age. Central and peripheral pulse pressures are independent risk factors for incident cardiovascular events in healthy individuals. Incident cardiovascular risk increases in aged individuals upon cold exposure via several mechanisms including cooling induced sympathetic nervous system activation and consequent vasoconstriction leading to elevated blood pressure and an increased cardiac afterload. These data suggest that in a healthy population spanning a wide age range, aortic pulse wave velocity is a stronger predictor of this increased risk relative to age. These data also suggest that a reduction of baseline aortic pulse wave velocity may attenuate the rise in central and peripheral pressor responses during any stressor, which presumably, elicits a sympatho‐excitatory response. Consequently, a lower baseline aortic pulse wave velocity and reduced pressor response during a stressor presumably eliciting an increased sympathetic activity may reduce the risk of acute incident cardiovascular events. Finally, aortic pulse wave velocity is one indices of arterial stiffness and the association reported here with central and peripheral pressor responses to the cold pressor test may vary depending upon the indices of arterial stiffness used (Lim et al. 2015).

## Conflict of Interest

There are no conflicts of interest to report.
